# Analgesic Effects of Ketamine, Magnesium Sulfate, and Sodium-Thiopental on Propofol Injection Pain: A Single-Blind Randomized Clinical Trial

**Published:** 2018-01

**Authors:** Hooshang Akbari, Ebrahim Nasiri, Attieh Nikkhah, Seyed Hossein Ardehali

**Affiliations:** 1 Department of Anesthesiology, Mazandaran University of Medical Sciences, Sari, Iran.; 2 Traditional and Complementary Medicine Research Centre, Mazandaran University of Medical Sciences, Sari, Iran,; 3 Antimicrobial Resistant Nosocomial Infection Research Center, Mazandaran University of Medical Sciences, Sari, Iran.,; 4 Department of Critical Care, Shohaday-e-Tajrish Hospital, Shahid Beheshti University of Medical Sciences, Tehran, Iran.

**Keywords:** Ketamine, Magnesium sulfate, Sodium-thiopental, Propofol, Injection, Pain, Analgesic

## Abstract

**Background:**

Propofol is one of the most frequently used medications for inducing and maintaining anesthesia. However, propofol injection causes pain and discomfort in more than 70% of patients. This study was performed to determine the comparative effects of ketamine, sodium-thiopental, and magnesium sulfate on reducing pain at the onset of anesthesia induced by propofol injection.

**Materials and Methods:**

This single-blind randomized clinical trial was conducted on a population of patients, requiring nonemergency surgeries. The sample size was determined as 25 patients per group. The eligible samples were randomly divided into three groups. An 18-gauge intravenous catheter was inserted in the dorsum of the hand for all patients. Three groups received 0.5 ml/kg of ketamine, 30 mg/kg of magnesium sulfate, and 0.5 ml/kg of sodium-thiopental, respectively. Next, 2.5 mg/kg of propofol 2% was administered at a rate of 1 ml/s. The verbal rating scale (VRS) was applied to assess the severity of pain during injection.

**Results:**

According to the results, the prevalence of pain was 36% in the magnesium sulfate group, 16% in the sodium-thiopental group, and 4% in the ketamine group. The ordinal logistic regression test showed that patients from the ketamine group experienced less pain, compared to the magnesium sulfate group (OR, 0.045; P= 0.008). However, no significant difference was observed between the ketamine and sodium-thiopental groups (OR, 0.253; P= 0.283).

**Conclusion:**

Ketamine and sodium-thiopental can be effective medications in reducing pain caused by propofol injection. According to the results, magnesium sulfate is not recommended for reducing pain due to propofol injection.

## INTRODUCTION

Propofol is one of the most common medications for inducing and maintaining anesthesia, with advantages such as rapid action and recovery. However, propofol injection causes pain and discomfort in more than 70% of patients (1). There are different strategies to prevent pain on propofol injection, including adjustment of injection speed, carrier fluid (2), solvent (3), and concomitant use of drugs.

Various drugs are currently used to relieve pain caused by propofol injection, such as lidocaine (1), tramadol and ondansetron (4), a combination of propofol and alfentanil (5), and a combination of propofol and lidocaine (6). Studies have shown that remifentanil is as effective as lidocaine in reducing pain caused by propofol injection (7). However, the side effects of these medications should be taken into consideration.

Ketamine is another drug with analgesic effects, used in anesthesia induction. It is an N-methyl-D-aspartate (NMDA) receptor antagonist with strong analgesic effects, even at low concentrations. Some studies have revealed that ketamine can significantly reduce the pain caused by propofol injection (8, 9). Magnesium sulfate is another drug used to reduce propofol-induced pain. It is a natural calcium antagonist, which can be used as an adjuvant agent to reduce postoperative pain (10–12). Various studies have shown that magnesium sulfate can reduce the need for opiates, such as propofol, remifentanil, and vacuronium (10, 13, 14). Another study compared the effects of magnesium sulfate and normal saline on propofol injection pain and showed greater pain reduction in the magnesium sulfate group, compared to the normal saline group (15).

Sodium-thiopental is another frequently used anesthetic agent. In this regard, the results of a study on children showed that a mixture of sodium-thiopental and propofol can lead to a greater reduction of pain, caused by propofol injection (16). Another study showed that concomitant use of sodium-thiopental and propofol can significantly reduce propofol injection pain, even with 10% sodium-thiopental (17).

The pain caused by propofol injection is still a common problem in anesthesia. Considering its extensive use and necessity of mitigating the associated complications, examination of various strategies seems essential.

The present study was conducted to determine the comparative effects of ketamine, sodium-thiopental, and magnesium sulfate on reducing propofol injection pain at the onset of anesthesia.

## MATERIALS AND METHODS

### Study design

This single-blind, parallel-design, randomized clinical trial was conducted on a population of patients, requiring nonemergency surgeries at Booali Hospital of Sari, Iran. The inclusion criteria were age range of 18–65 years and class I and II ASA, while the exclusion criteria were highly increased intracranial pressure, unstable hemodynamics, severe mental disorders, history of allergy to drugs, kidney, liver, or heart dysfunction, addiction, and use of calcium or magnesium blockers.

### Sample size

Considering 90% confidence interval, 80% power, and 30% difference based on a previous study (18), the sample size was determined as 25 patients for each group. After obtaining approval from the Ethics Committee of Mazandaran University of Medical Sciences and collecting informed consents from the patients, eligible samples were randomly divided into three groups.

### Intervention

Before the intervention, an 18-gauge intravenous catheter was inserted in the dorsum of the hand for all patients. One minute prior to propofol injection, all patients received the study drug through a 5 mL syringe. In the ketamine group, the patients received 0.5 ml/kg of ketamine (production series, 11-207; Trittau, Germany) (18). In the magnesium sulfate group, magnesium sulfate (production series, 1228054891; Pasteur Institute, Iran) was administered at a concentration of 30 mg/kg (10, 12–14), and in group three, the patients received 0.5 ml/kg of sodium-thiopental (19) (production series, 207; Trittau, Germany) at a rate of 1 mg/sec.

Next, 2.5 mg/kg of propofol 2% (production series, 34209; Melsungen, Germany) was administered at a rate of 1 ml/sec. The verbal rating scale (VRS) was immediately used after injection of drugs to assess the severity of pain during injection (20–22). It involved facial expressions, withdrawal of the hand, tears, and complaints about pain (further explanation required, particularly about score rating).

In the VRS, “none” describes patients who experience no pain; “mild pain” describes patients who respond to feelings of pain when questioned, but have no facial grimacing and do not cry; “moderate pain” describes patients who have facial grimacing, show withdrawal of the hands, respond positively to feelings of pain, or complain of pain spontaneously; and “severe pain” describes patients who voluntarily complain of pain, have facial grimacing, and show withdrawal of the hands (20).

The proper concentration of propofol was injected to complete anesthesia induction. For muscle relaxation, 0.5 mg/kg of atracurium was administered. Oral intubation was performed, and anesthesia was maintained with isoflurane and 50% nitrous oxide and oxygen. The collected data were analyzed in SPSS version 20, using descriptive statistics (mean, percentage, and standard deviation) and analytical tests (ordinal logistic regression) at a significance level of *P*< 0.05.

### Randomization

After obtaining approval from the Ethics Committee of Mazandaran University of Medical Sciences and collecting written informed consents from the patients, they were assured of the confidentiality of data. The eligible participants were then given a brief description about the study. The participants were randomly divided into three groups via block randomization method. Recruitment of participants was sequential in the study. The samples were also blind to the type of intervention.

### Statistical analysis

The severity of pain was evaluated with VRS and compared between the groups, using the ordinal logistic regression model. Numerical data are presented as mean and standard deviation. Data were analyzed in SPSS version 20, and the significance level was set at *P*< 0.05.

## RESULTS

**Figure 1.**
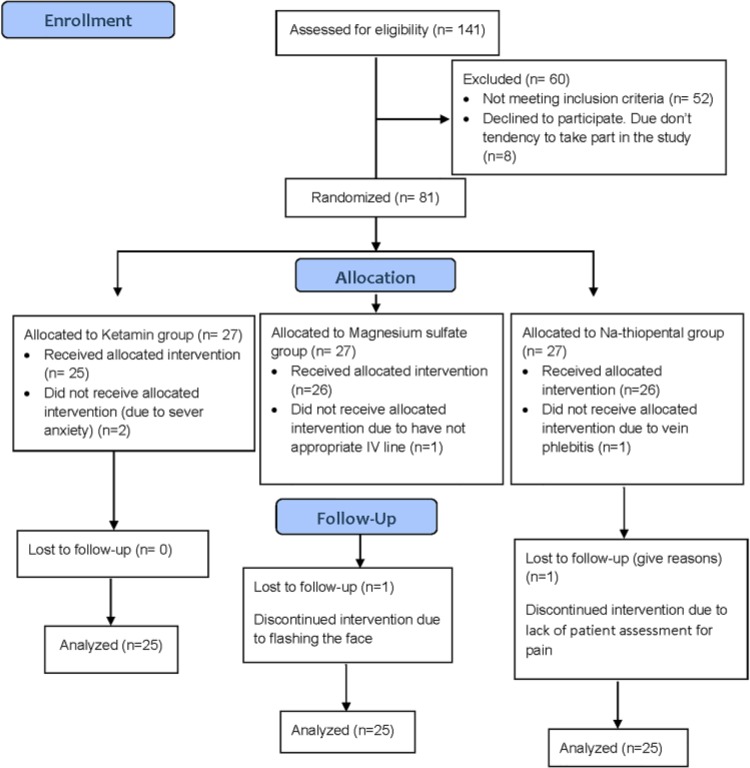
CONSORT Flow Diagram of the study

A total of 71 patients were recruited in this study (27 patients per group). Two patients were excluded from the ketamine group due to severe anxiety. Also, two patients were eliminated from the magnesium sulfate group due to lack of an appropriate intravenous line and face flashing. Moreover, phlebitis and lack of pain assessment resulted in the exclusion of patients from the sodium-thiopental group (Figure 1). The mean age of patients in the ketamine, magnesium sulfate, and sodium-thiopental groups was 31.32±13.4, 34±10.7, and 35.8±12.9 years, respectively. The majority of patients from the three groups had ASA class I. Table 1 presents the demographic characteristics of the patients.

**Table 1.** Patient characteristics. Values are mean (SD), median (IQR), or number of patients.

	**Ketamin** (n=25)	**Magnesium sulfate** (n=25)	**Na-thiopental** (n=25)
**Gender (M/F)**	11/14	13/12	14/11
**Age (yr)**	31.32±13.406 (18–60)	34±10.74 (18–57)	35.8± 12.909 (22–70)
**Weight (kg)**	63.36±11.34 (40–85)	65.88±13.5 (30–95)	69.2± 17.806 (50–140)
**ASA (I/II)**	24/1	24/1	17/8

According to the results, the prevalence of pain was 36% in the magnesium sulfate group, 16% in the sodium-thiopental group, and 4% in the ketamine group. Table 2 presents the number of patients, who still reported pain after administration of solutions. According to the ordinal logistic regression test, patients from the ketamine group experienced less pain, compared to the magnesium sulfate group (OR, 0.045; *P*= 0.008). However, no significant difference was observed between the ketamine and sodium-thiopental groups (OR, 0.253; *P*= 0.283) (Table 3).

**Table 2.** Assessment of pain during iv injection of propofol

**Pain N(%)**	**Ketamin** (n=25)	**Magnesium sulfate** (n=25)	**Na-thiopental** (n=25)
**No pain**	24(96%)	16(64%)	21(84%)
**Mild**	1(4%)	4(16%)	1(4%)
**Moderate**	-	5(20%)	1(4%)
**Severe**	-	-	2(8%)

**Table 3.** Comparison of pain in the three groups under study

**Parameter**	**Ordinal Logistic Regression**				
		OR*	**95% Confidence Interval for OR**		**P value**
			**Lower**	**Upper**	
**Drug Group**	Na-thiopental	0.253	0.020	3.112	0.283
	Mg Sulfate	0.045	0.004	0.448	0.008
	Ketamin**	1	-	-	-

* Adjusted for sex, age and BMI.

** Reference level.

## DISCUSSION

Despite the lack of a significant difference in pain between the ketamine and sodium-thiopental groups, the ketamine group reported less pain. The highest level of pain was reported by patients from the magnesium sulfate group. In agreement with the results of the present study, Saadawy et al. compared the effects of ketamine (0.4 mg/kg), sodium-thiopental (0.5 mg/kg), pethidine (0.4 mg/kg), lidocaine (1 mg/kg), and normal saline (3 mL) and found the prevalence of pain to be 8% and 32% in the ketamine and sodium-thiopental groups, respectively. The findings showed that administration of ketamine prior to propofol injection is the best strategy to reduce pain caused by propofol injection (17).

Özkoçak et al. compared the effects of 2 mL of normal saline, 70 μg/kg of epinephrine, and 0.5 mg/kg of ketamine on pain caused by propofol injection and found no significant difference in the incidence of pain between the groups; however, the severity of pain was significantly different between the ketamine and other groups (18). In their study, a numerical scale was used to assess the severity of pain.

In addition, Zahedi et al. compared the effects of three different doses of ketamine on pain caused by propofol injection. They divided the patients into five groups, receiving 1 mg/kg of lidocaine, normal saline, and ketamine at concentrations of 50, 75, and 100 μg/kg, respectively. The findings showed a significant difference in the incidence and severity of pain between the normal saline (placebo) and other groups. The incidence and severity of pain were lower in the 100 μg/kg ketamine group, compared to the 50 μg/kg ketamine and lidocaine groups (24).

Previous studies have shown that the analgesic effects of low ketamine doses can complement the sedative effects of propofol injection (25). Combination of ketamine and propofol seems to produce greater sedative effects and limited toxicity. This mixture produces greater analgesic effects, compared to propofol alone, improves spontaneous breathing, and accelerates postoperative recovery (26).

The results of the present study showed no significant difference in pain between the sodium-thiopental and ketamine groups. Overall, many studies have examined the effects of sodium-thiopental prior to propofol injection. In this regard, Agarwal et al. compared the effects of normal saline, lidocaine 2% (4 mg), and sodium-thiopental at doses of 0.5 and 0.25 mg/kg on pain caused by propofol injection. Pain-related complaints were reported in 77% of patients from the saline group, 39% of patients from the lidocaine 2% group, 32% of patients from the 0.5 mg/kg sodium-thiopental group, and 3% of patients from the 0.25 mg/kg sodium-thiopental group. Accordingly, administration of 0.5 mg/kg of sodium-thiopental was recommended one minute before propofol injection (23).

Additionally, Pollard et al. examined the effects of sodium-thiopental on propofol injection pain among children. The patients were divided into propofol plus lidocaine and propofol plus sodium-thiopental groups. According to the results, the incidence of pain was 14% in the propofol plus sodium-thiopental group and 35% in the propofol plus lidocaine group; a significant difference was observed between the groups (16). Although this study was conducted on children, the results are similar to the present study in terms of sodium-thiopental efficacy. It can be concluded that sodium-thiopental leads to the relative control of pain caused by propofol injection in both children and adults.

In the present study, the magnesium sulfate group reported greater pain, compared to other groups. In contrast, Memis et al. in their report showed that magnesium sulfate can reduce the pain due to propofol injection. In their study, the patients were divided into normal saline and magnesium groups. The results showed a significant difference in pain scores between the groups (15). In contrast to the present study, normal saline was compared with magnesium sulfate, while in agreement with the present study, 36% of patients reported pain in the magnesium group.

In another study, Agarwal et al. showed that intravenous magnesium and lidocaine were equally effective in reducing pain caused by propofol injection. However, administration of magnesium in isolation appears to induce pain, and its use cannot be justified (27).

There are some potential limitations in the present study. If pain assessment was combined with other techniques, it would have become more accurate. Also, further use of lidocaine, as the gold standard for propofol pain reduction, needs to be considered. Another limitation of this study was our inability to assign uniformed nursing staff to intravenous catheter insertion procedures; this might have in fact limited our control over confounding variables.

## CONCLUSION

Ketamine and sodium-thiopental can be ideal medications for reducing pain caused by propofol injection. However, considering its analgesic and sympathomimetic properties, ketamine appears to be a more suitable agent. According to the results, magnesium sulfate is not recommended for reducing the pain caused by propofol injection. Further studies are required to confirm the external validity of the results.
